# Risk of venous thromboembolism in Asian patients with inflammatory bowel disease: a nationwide cohort study

**DOI:** 10.1038/s41598-021-81657-y

**Published:** 2021-01-21

**Authors:** Chan Mi Heo, Tae Jun Kim, Eun Ran Kim, Sung Noh Hong, Dong Kyung Chang, Mi Yang, Seonwoo Kim, Young-Ho Kim

**Affiliations:** 1 Division of Gastroenterology, Department of Medicine, Samsung Medical Center, Sungkyunkwan University School of Medicine, 81 Irwon-ro, Gangnam-gu, Seoul, 06351 Korea; 2 Statistics and Data Center, Samsung Medical Center, Sungkyunkwan University School of Medicine, Seoul, Korea

**Keywords:** Crohn's disease, Ulcerative colitis, Inflammatory bowel disease

## Abstract

Routine prophylaxis for venous thromboembolism (VTE) in Asian IBD patients has been controversial. We aimed to estimate the risk of VTE of Asian patients at different phases of IBD by incorporating patient-specific risk factors. In this cohort study, we analyzed the National Health Insurance claims data between 2012 and 2016 for the entire Korean population. We calculated incidence rates and hazard ratios for VTE. The overall VTE risk was higher in patients with IBD [adjusted hazard ratio (aHR), 2.06; 95% confidence interval (CI), 1.66–2.55], than in controls. When we compare the risk of VTE by different disease phases, the risk of VTE was the highest during post-operation period after IBD-related bowel surgery (aHR, 39.7; 95% CI 9.87–159.3), followed by during hospitalized periods with flare (aHR, 27.2; 95% CI 14.9–49.65) and during hospitalized periods with non-flare (aHR, 16.23; 95% CI 10.71–24.58). The incidence rate (per 1000 person-years) was 15.26 during hospitalized periods with a flare and 9.83 during hospitalized periods with non-flare. According to age groups, the incidence rate (per 1000 person-years) during hospitalized periods with flare was 14.53 in young patients (20–39 years) and 34.58 in older patients (60–80 years). During hospitalized periods with non-flare, the incidence rate was 3.55 in young patients and 23.61 in older patients. The prophylaxis of VTE for Asian patients with IBD should be recommended in older patients admitted to hospital and be considered in young patients who are hospitalized with a flare.

## Introduction

Deep vein thrombosis (DVT) and acute pulmonary embolism (PE) are most common manifestations of venous thromboembolism (VTE), which is a serious extraintestinal manifestation of inflammatory bowel disease (IBD) that can lead to substantial morbidity and mortality^1–6^. Thromboembolic events are potentially avoidable with thrombo-prophylaxis. However, prophylaxis is not free from cost burden and risk of side effects. Considering the risk or benefit of VTE prevention, prophylaxis is justified by the size of thromboembolic risk in hospitalized IBD patients with flares without severe bleeding^7^. In Western countries, VTE prophylaxis during hospitalization with flares is considered an indicator of the qaulity of care^8,9^.

Previous reports regarding the risk of VTE in IBD patients used data sources from Western countries, which have a high incidence of IBD. Recently, the incidence of IBD has been rising in South Korea, Japan, Taiwan, and China, areas in which the incidence was previously thought to be low^10–14^. Consensus or guidelines for VTE prophylaxis in Western population were well established, whereas prophylaxis for VTE in Asian countries has not been routinely performed. Indeed, only < 25% of East Asian gastroenterologists provide adequate prophylaxis for VTE^15^. In addition, recent multi-national study from East Asia suggested that close monitoring should be preferred over routine prophylaxis of VTE because the absolute incidence of VTE is lower among East Asian patients than Western population^16^. However, the previous study is based on overall risk of VTE, and does not stratify based on specific risk factors, including active disease state, hospitalization, recent surgery, and other co-morbidities. Therefore, we aimed to estimate the risk of VTE during the different disease phases of hospital admission, flares, and post-operation in a nationwide, population-based cohort of South Korea.

## Results

**Figure 1:**
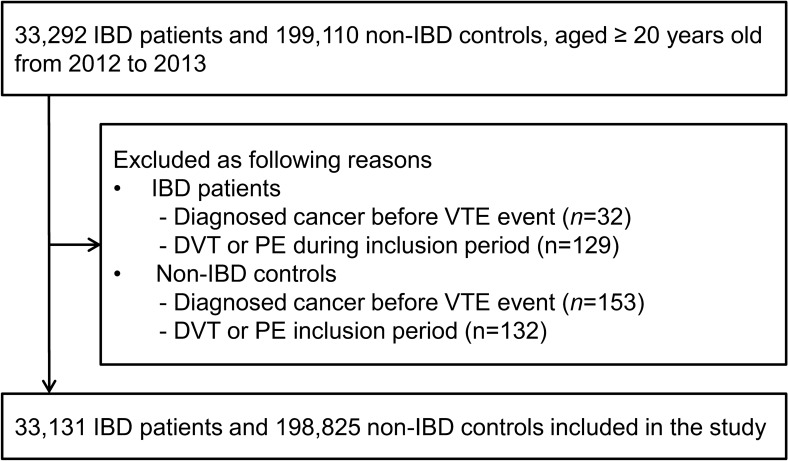
Flow chart of study participants.

During the inclusion period between January 1, 2012 and December 31, 2013, we identified 33,292 IBD patients and 199,110 non-IBD controls. Among them, 185 patients were diagnosed with cancer before the VTE event and 261 patients developed DVT or PE during inclusion period. Therefore, 33,131 patients with IBD and 198,825 non-IBD controls were included in the cohort (Fig. 1). The demographic and clinical characteristics of the study population are presented in Table 1. The percentages of each age group and sex were the same between the patients and controls. Thirty-nine percent of patients were women, and the median age at cohort entry was 42 years. A history of co-morbidities was less likely in IBD patients than in controls (*P* < 0.001). Control group were more likely to have a history of hypertension, diabetes mellitus, and cardiovascular disease.

**Table 1:** Characteristics of study population, IBD patients versus controls.

	IBD patients	Controls	*P* value
Number	33,131	198,825	
**Age category (%)**			
20–29	20.7	20.7	
30–39	21.9	21.9	
40–49	21.1	21.1	
50–59	18.9	18.9	
60–69	10.8	10.8	
70–80	6.6	6.6	
**Sex (%)**			
Male	60.7	60.7	
Female	39.3	39.3	
**Disease phenotype (%)**			
Crohn’s disease	29.9		
Ulcerative colitis	70.0		
Unspecified	0.1		
Co-morbidities (%)	23.0	26.2	< 0.001
Hypertension	17.2	20.8	< 0.001
Diabetes Mellitus	11.5	13.8	< 0.001
Cardiovascular disease	5.1	6.3	< 0.001
Cerebrovascular disease	2.8	2.9	0.087
Heart failure	1.4	1.5	0.283
Atrial fibrillation	0.8	0.8	0.249
Chronic kidney disease	1.0	0.9	0.027
**Medications (ever use, %)**			
5-ASA	90.5		
Corticosteroids	59.2		
Immunomodulators	29.2		
Anti-TNF-α agents	8.6		

Values are expressed as means ± standard deviation or percentages.

*IBD* inflammatory bowel disease; *5-ASA* 5-aminosalicylic acid; *Anti-TNF-α* anti-tumor necrosis factor α.

The number of events and incidence rates of VTE, DVT, and PE in IBD patients and the matched controls are shown in Table 2. During the 99,206 person-years follow-up, VTE developed in 110 patients with IBD. During the 595,912 person-years follow-up, VTE developed in 376 non-IBD controls. The incident rates (per 10,000 person-years) of VTE were 11.1 for IBD patients and 6.3 for non-IBD controls. The incidence of each event increased with age in both groups and was higher in female than in male patients. Overall, the risk of VTE was about twofold higher in patients with IBD than in controls (adjusted HR, 2.06; 95% CI 1.66–2.55) When we further adjusted for a history of anticoagulation therapy, the risk reduced, however, it was still evident (adjusted HR, 1.78; 95% CI 1.42–2.23). (Table 3). Risks were higher for patients with CD, compared to those with UC (adjusted HR, 4.43; 95% CI 2.67–7.34 vs. adjusted HR, 1.74; 95% CI 1.31–2.32). Female patients with IBD had a slightly higher risk of VTE than male patients with IBD (adjusted HR, 2.66; 95% CI 1.89–3.74 vs. adjusted HR, 1.64; 95% CI 1.14–2.34). We also evaluated if the risk of VTE in IBD patients differed in pre-specified subgroups defined by age, sex, disease phenotype, and co-morbidities. The elevated risk of VTE in IBD patients was consistently observed in all subgroups analyzed (Supplementary Table S1).

**Table 2:** Incident cases and incident rates per 10,000 person-years of venous thromboembolism, deep vein thrombosis, and pulmonary embolism in IBD patients and non-IBD controls.

	IBD (N = 33,131)			Non-IBD (N = 198,825)		
	Incident cases	Person-years	Incident rate	Incident cases	Person-years	Incident rate
**VTE**						
Overall	110	99,206	11.1	376	595,912	6.3
Sex						
Male	49	60,268	8.1	209	361,824	5.8
Female	61	38,938	15.7	167	234,087	7.1
Age group						
20–39	19	42,319	4.5	33	254,058	1.3
40–59	27	39,692	6.8	103	238,389	4.3
60–80	64	17,195	37.2	240	103,466	23.2
**DVT**						
Overall	78	99,253	7.9	215	596,128	3.6
Sex						
Male	36	60,288	6.0	112	361,955	3.1
Female	42	38,965	10.8	103	234,174	4.4
Age group						
20–39	14	42,324	3.3	17	254,075	0.7
40–59	21	39,697	5.3	55	238,454	2.3
60–80	41	17,231	23.8	143	103,599	13.8
**PE**						
Overall	32	99,333	3.2	161	596,221	2.7
Sex						
Male	13	60,328	2.2	96	361,993	2.6
Female	19	39,005	4.9	65	234,228	2.8
Age group						
20–39	5	42,340	1.2	18	254,076	0.7
40–59	6	39,732	1.5	52	238,463	2.2
60–80	23	17,261	13.3	91	103,682	8.8

*IBD* inflammatory bowel disease; *VTE* venous thromboembolism; *DVT* deep vein thrombosis; *PE* pulmonary embolism.

**Table 3:** Risk of venous thromboembolic events in patients with IBD.

	Crude model*		Model 1**		Model 2***		Model 3****	
	HR (95% CI)	*P* value	HR (95% CI)	*P* value	HR (95% CI)	*P* value	HR (95% CI)	*P* value
VTE	1.76 (1.42–2.17)	< 0.001	1.96 (1.58–2.43)	< 0.001	2.06 (1.66–2.55)	< 0.001	1.78 (1.42–2.23)	< 0.001
DVT	2.14 (1.67–2.75)	< 0.001	2.47 (1.92–3.19)	< 0.001	2.58 (2.00–3.33)	< 0.001	2.20 (1.68–2.88)	< 0.001
PE	1.23 (0.85–1.77)	0.267	1.30 (0.90–1.88)	0.159	1.39 (0.96–2.00)	0.081	1.27 (0.87–1.86)	0.217

Reference: non-IBD controls.

*IBD* inflammatory bowel disease; *VTE* venous thromboembolism; *DVT* deep vein thrombosis; *PE* pulmonary embolism; *HR* hazard ratio; *CI* confidence interval.

*Matched on age and sex.

**Multivariable-adjusted model 1: adjustment for provoking factors (recent surgery, fracture, systemic corticosteroids, and pregnancy within 90 days of event).

***Multivariable-adjusted model 2: Model 1 plus adjustment for co-morbidities (hypertension, diabetes, cardiovascular disease, cerebrovascular disease, heart failure, atrial fibrillation, and chronic kidney disease).

****Multivariable-adjusted model 3: Model 2 plus adjustment for history of anticoagulation therapy.

**Figure 2:**
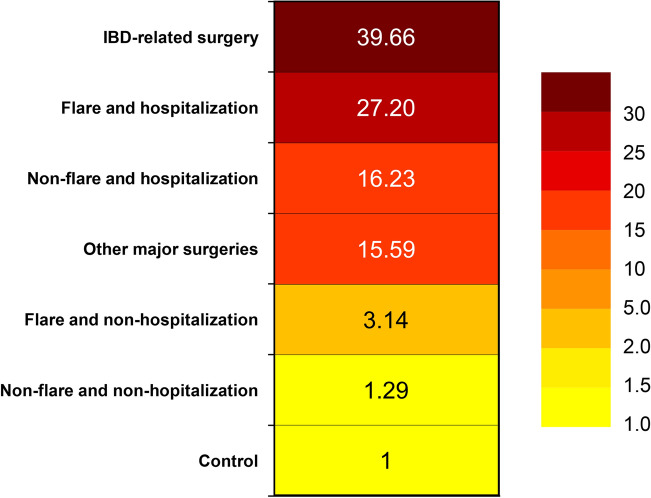
Heat map of risk of venous thromboembolism by different phases of IBD patients. Adjustment for age, sex, provoking factors (recent surgery, fracture, and pregnancy within 90 days of event), and co-morbidities.

The incidence rates of VTE among IBD patients varied greatly depending on the period of disease activity, hospitalization, and surgery (Table 4). The heat map of risk of VTE at different phases in patients with IBD is shown in Fig. 2. The color intensity of the heat map is based on the HRs for the VTE. After adjustment for age, sex, provoking factors (recent surgery, fracture, and pregnancy within 90 days of event), and co-morbidities, the risk of VTE was about fivefold higher at the time of a flare (adjusted HR, 4.68; 95% CI 3.14–6.99), and was slightly increased during inactive state (adjusted HR, 1.75; 95% CI 1.38–2.22). According to the disease activity and hospitalization, the risk of VTE during a non-hospitalized flare in IBD patients was higher than in controls (adjusted HR, 3.14; 95% CI 1.90–5.19). The risks of VTE were greatly increased during a hospitalization with non-flare (adjusted HR, 16.23; 95% CI 10.71–24.58) and a hospitalization with flare (adjusted HR, 27.2; 95% CI 14.9–49.65). The median duration (interquartile range) until VTE development were 24 (2–69) days for a hospitalization with flares, 34 (21–53.5) days for a hospitalization without flare, 40 (18.5–102.5) days for a non-hospitalized flare, and 113.5 (26–267) days for non-hospitalization and non-flare. Finally, with respect to surgery, the risk of VTE was highest at the time of IBD-related surgery (adjusted HR, 39.66; 95% CI 9.87–159.33). The risk at the time of other major surgery was also increased (adjusted HR, 15.59; 95% CI 7.73–31.43).

**Table 4:** Incidence of venous thromboembolism by disease activity, hospitalization, and surgery.

	Events (n)	Person-years	Incidence (per 1000 person-years)
**Disease activity**			
IBD	110	99,206.2	1.11
Flare	27	9263.4	2.81
Inactive (non-flare)	83	89,942.8	0.92
Control	376	595,911.7	0.63
**Disease activity and hospitalization**			
IBD	110	99,206.2	1.11
Flare and hospitalization	11	720.7	15.26
Flare and non-hospitalization	16	8922.5	1.79
Non-flare and hospitalization	24	2441.3	9.83
Non-flare and non-hospitalization	58	87,121.8	0.67
Control	376	595,911.7	0.63
**Surgery**			
IBD	110	99,206.2	1.11
IBD-related surgery	2	152.3	13.13
Other major surgery	8	626.6	12.77
Non-surgical period	100	98,427.3	1.02
Control	376	595,911.7	0.63

In the IBD cohort, the risk of VTE was greatly increased during a hospitalized flare (adjusted HR, 25.49; 95% CI 13.11–49.56) and a hospitalization without flare (adjusted HR, 13.52; 95% CI 8.33–21.96) than during a non-flare and non-hospitalization. In a non-hospitalized flare, the risk of VTE was slightly increased (adjusted HR, 2.83; 95% CI 1.60–5.03). The magnitude of this finding was similar to the results obtained by the Cox regression analysis with a different reference category (Table 5).

**Table 5:** Risk of venous thromboembolism by disease activity and hospitalization in IBD cohort.

	Incidence (per 1000 person-years)	Crude HR (95% CI)	P value	Adjusted HR* (95% CI)	P value
**Disease activity and hospitalization**					
Flare and hospitalization	15.26	26.4 (13.66–51)	< 0.001	25.49 (13.11–49.56)	< 0.001
Non-flare and hospitalization	9.83	15.27 (9.44–24.69)	< 0.001	13.52 (8.33–21.96)	< 0.001
Flare and non-hospitalization	1.79	3.12 (1.77–5.52)	< 0.001	2.83 (1.60–5.03)	< 0.001
Non-flare and non-hospitalization	0.67	1.00 (reference)		1.00 (reference)	

*IBD* inflammatory bowel disease; *HR* hazard ratio; *CI* confidence interval.

*Adjustment for age, sex, provoking factors (recent surgery, fracture, and pregnancy within 90 days of event), and co-morbidities.

Finally, we calculated the incidence rate (per 1000 person-years) by disease activity and hospitalization according to age groups (Young patients aged 20–39 years old vs. older patients aged 60–80 years old) (Table 6). The incidence rate during hospitalization with flares was 14.53 in young patients and 34.58 in older patients. At the time of hospitalization with non-flare, the incidence rate was 3.55 in young patients and 23.61 in older patients. During ambulatory flares, the incidence rate was 0.58 in young patients and 6.64 in older patients.

**Table 6:** Incidence of venous thromboembolism by disease activity and hospitalization according to age groups (young vs. old).

Disease activity and hospitalization	Events (n)	Person-years	Incidence (per 1000 person-years)
**Young patients with IBD (20–39 years old)**	19	42,319.5	0.45
Flare and hospitalization	5	344	14.53
Flare and non-hospitalization	3	3464	0.58
Non-flare and hospitalization	4	1126.5	3.55
Non-flare and non-hospitalization	8	37,385	0.21
**Older patients with IBD (60–80 years old)**	64	17,194.5	3.72
Flare and hospitalization	5	144.6	34.58
Flare and non-hospitalization	11	1657.7	6.64
Non-flare and hospitalization	12	508.2	23.61
Non-flare and non-hospitalization	36	14,884	2.42

## Discussion

To our knowledge, this is the first Korean population-based analysis of VTE risk in IBD. We found that patients with IBD had about a twofold higher risk of VTE than in a non-IBD population. In particular, the risk of VTE varied greatly depending on the age, disease activity, hospitalization, and surgery in patients with IBD. Compared with the non-IBD population, the risk of VTE was increased about 27-fold during hospitalization with IBD flares. Patients who underwent IBD-related bowel resection had about a 40-fold higher risk of VTE during postoperative period. Despite the low absolute risk of VTE in Asian patients with IBD, these results suggest that prophylaxis should be considered in hospitalized patients with active disease state.

Few studies have previously examined the risk of VTE in Asian patients with IBD^16–19^. Most of the studies have focused on the overall risk of VTE in IBD patients compared with the general population. A recent multinational retrospective study of 2562 hospitalized IBD patients reported that Asian patients with IBD carry a twofold increased risk of VTE than the general population^16^. Because the absolute risk of VTE in Asian patients (1.15 per 1000 person-years)^16^ is lower than that observed in Western patients (2.6 per 1000 person-years)^7^, the study recommended close monitoring rather than routine prophylaxis of VTE in Asian patients with IBD. However, the study did not contain information regarding outpatient, or inpatient periods, disease activities, and other provoking factors such as surgeries. In this study, the risk of VTE varied greatly depending on the disease activity, hospitalization, and major surgery in patients with IBD and hospitalized patients experiencing disease flares carry a 19-fold increased risk of VTE compared with non-IBD population. Another key finding in this study was the strong effect of patient age on VTE risk. In IBD patients aged 40 or younger, the absolute incidence rate of VTE was very low approximately 4.5 events per 10,000 person-years. However, in older patients with IBD, aged ≥ 60, the annual incidence rate of VTE was 37.2 events per 10,000 person-years.

The actual incidence of VTE in different phases of IBD activity may be more appropriate to support decision-making on prophylaxis against VTE. The overall incidence of VTE in Asian patients with IBD found in this study was lower than that of Western patients reported in previous studies (1.11 vs. 2.4 and 2.6 per 1000 person-years)^7,20^. However, the incidence of VTE during hospitalization with flares was greatly increased to 15.26 (per 1000 person-years). The incidence rate in older patients (60–80 years) was further increased to 34.58 during hospitalization with flares and 23.61 during hospitalization with non-flare. The incidence rate of VTE during hospitalization with flares in older patients was nearly identical to that reported by previous study in the UK (34.6 vs. 37.5 per 1,000 person-years)^7^; however, the incidence rate of young patients during hospitalization with flares was somewhat lower (14.53 per 1,000 person-years). Considering both absolute risk as well as relative risk in the recommendations for VTE prophylaxis, the prophylaxis of VTE for Asian patients with IBD should be recommended in older patients admitted to hospital and be considered in young patients who are hospitalized with flares.

Major surgery in patients aged ≥ 40 years is a moderate to high risk for VTE and recent guideline recommends VTE prophylaxis for these patients^21^. In the present study, IBD patients after IBD-related major surgery had a 40-fold increased risk than the baseline. The risk at the time of other major surgery also had a 15-fold increase. In Asian patients with IBD, major surgery, especially IBD-related surgery, is an important risk factor for VTE and prophylaxis should be considered.

The primary strength of this study is the nationwide population-based design, providing a cohort covering the entire Korean population with IBD with minimal loss to follow-up. The validation of the diagnosis of IBD using RID has been performed in previous studies. In a study on IBD epidemiology, Kim et al. reported that a chart review of 800 medical records from 8 hospitals demonstrated a 97.3% sensitivity and 92.6% specificity for UC, and 98.3% sensitivity and 93.5% specificity for CD^11^. In other studies, a high level of agreement between hospital and administration data was also reported^22,23^. Furthermore, we believe that our outcome definition (medical code relating to DVT or PE along with a pharmacy claim for anticoagulants) was more accurate than an outcome definition that includes only a medical code.

This nationwide population-based study in Asia provides useful information about VTE risk in Asian patients with IBD. Nonetheless, there are several limitations to this study. First, the possibility of misclassification was not excluded because we used insurance claim data. Particularly, our definition of active disease might deserve more scrutiny. This study defined acute flare as the use of new systemic corticosteroids or anti-TNF agents, an imprecise surrogate indicator of active disease. Because systemic corticosteroids or anti-TNF agent are typically used for the treatment of moderate to severe IBD, we could interpret acute flare to include those of at least moderate to severe disease activity and not mild flares. We have assumed that a flare leaves all patients at risk for 120 days, whereas the actual duration of flare would vary from individual to individual. The analysis of the cohort using chart review or electronic medical record data with more precise measurement of severity and duration of flare would be advantageous. Third, in the detection of VTE, there is possibly a surveillance bias linked to the better monitoring in IBD patients than in the non-IBD population. Finally, although we measured several important confounders in the multivariable analysis, we cannot exclude the possibility of residual confounding factors due to unmeasured parameters not included in the Korean National Health Insurance claims data, including laboratory data, behavioral factors such as smoking and alcohol intake, anthropometric characteristics such as body mass index, over the counter medication use such as oral contraceptives, and hormonal replacement therapy. However, by analyzing large-scale administrative data from the entire Korean population, the risk estimates were only mildly attenuated.

This study showed that the risk of VTE was higher in Korean patients with IBD than in non-IBD population. The results from this study provide strong evidence that the risk of VTE varies greatly by patient age, disease activity, hospitalization, and major surgery. Due to the low absolute risk of VTE in Asian patients with IBD, our results suggest that there is a need to modify the currently recommended ‘one-size-fits-all’ approach to recommendations regarding VTE prophylaxis. We believe that physicians in Asian countries should take into account patient-specific estimates of absolute risk as well as current recommended guidelines of VTE prophylaxis. In Asian patients with IBD aged less than 60 year, the absolute risks of VTE are considerably lower. In these younger patients without other risk factors for VTE, the benefits of routine prophylaxis may no longer outweigh the risks. Young, hospitalized patients experiencing disease flares might benefit from VTE prophylaxis. The prophylaxis of VTE should be recommend for older patients (> 60 years of age) who were admitted to hospital. We also urge physicians to recognize the markedly increased risk in Asian patients after IBD-related surgery or other major surgery and consider VTE prophylaxis for these patients.

## Methods

### Data sources

We carried out a cohort study, analyzing the inpatient and outpatient insurance claims data in the Health Insurance and Review Agency (HIRA) database for the 5–year period from January 2012 to December 2016. The National Health Insurance (NHI) program of South Korea is a universal health coverage system that covers the inpatient, outpatient, and pharmaceutical service expenditures of the entire Korean population comprising approximately 51 million people. All healthcare information is registered in a comprehensive database operated by the HIRA, and reimbursement applications are reviewed on the basis of this data. The claims data of the HIRA includes the demographic characteristics of patients, diagnosis using the International Classification of Disease 10th revision (ICD-10), treatments, procedures, surgical history, and prescribed drugs^24^. In addition, the Korean government established a registration program for Rare Intractable Diseases (RID) that includes serious and costly long-term diseases, including Crohn’s disease (CD) and ulcerative colitis (UC). The RID registration program provides enhanced reimbursement for medical expenses related to the conditions it covers. Therefore, patients with IBD are registered using a uniform and definite criteria, after being diagnosed^11^. This study was approved by the Institutional Review Board of the Samsung Medical Center was conducted in accordance with the Declaration of Helsinki. The Institutional Review Board also approved that requirement for informed consent was waived since we used only de-identified data collected from the HIRA.

### Study population

All patients aged 20 to 80 years and diagnosed as having IBD from January 2012 to December 2016 were included in this study and were followed up until December 2016. Patients registered as having IBD in the HIRA claims database (ICD-10 codes K50.0–50.9 and special co-payment program code V130 for CD; ICD-10 codes K51.0–51.9 and special co-payment program code V131 for UC) were included in this study. An individual was considered to have IBD if he or she had 1 hospital discharge diagnosis including K50 or K51 along with a pharmacy claim for an IBD medication. IBD medications were categorized as 5-aminosalicylic acid ([ASA] including mesalamine and sulfasalazine), immunomodulators (including azathioprine, mercaptopurine, and methotrexate), and anti-tumor necrosis factor (TNF)-α (including infliximab and adalimumab). The other biologic agents such as vedolizumab were not used in Korea until 2016. Non–IBD controls were used to represent the general population. For each case, we randomly selected up to six non–IBD controls matched on the basis of age and sex corresponding to one case. Using the same method, the controls for CD and UC were also selected from the non-IBD population. Controls did not have IBD. We excluded the patients with a diagnosis of VTE during the inclusion period, between January 2012 and December 2013. Individuals with malignancy were excluded as it is a major risk factor for VTE.

### Exposure definition

The risk of VTE is associated with disease activity, recent hospitalization or surgery. We used a new corticosteroid or anti-TNF agent prescription as a surrogate measure for an acute flare or active disease. A flare or active disease period was considered as lasting during 120 days following the first prescription of drug after at least 4 months without such a prescription. Patient, who were admitted hospital or underwent surgery, was considered as being exposed during 90 days following the date of admission or surgery. At each time of the follow-up, patients were categorized as being exposed to active disease (flare) period, hospitalization, surgery, or combination of those. A hospitalized flare was defined as new corticosteroid or anti-TNF agent prescription with hospitalization for an event related to the symptoms of IBD and the patients were considered as being exposed during the period of 120 days. If a patient was hospitalized within the flare period of 120 days, we defined a flare hospitalization. A non-flare hospitalization was defined as hospitalization without new corticosteroid or anti-TNF agent prescription and the patients were considered as being exposed during the period of 90 days. Surgeries were classified into IBD-related surgery, such as bowel resection, and other major surgeries.

### Outcomes

The main outcome was the development of VTE during the follow-up period until December 2016. In the analysis of the primary outcome, we excluded the patients with VTE during the inclusion period, between January 2012 and December 2013. VTE was defined as ICD-10 codes (Supplementary Table S2) related to the diagnosis of deep vein thrombosis (DVT) or pulmonary embolism (PE) along with a pharmacy claim for anticoagulants, including heparin, low-molecular weight heparin, warfarin, and direct oral coagulants such as rivaroxaban, dabigatran, or apixaban within 1 month of the date of diagnosis. The follow-ups were conducted until December 2016 or censored at the date of diagnosis of VTE.

### Covariates

Time-fixed covariates were defined at cohort entry and included age, sex, and IBD type. History of hypertension, diabetes mellitus, cardiovascular disease, cerebrovascular disease, heart failure, atrial fibrillation, and chronic kidney disease related conditions defined based on ICD-10 codes were included as potential confounding covariates. In addition, recent surgery, bone fracture, and pregnancy within 90 days of the event were included as common provoking factors of DVT. We also included history of systemic corticosteroid and anticoagulation therapy as potential confounders in the multivariable analysis (Table 3).

### Statistical analysis

Categorical variables were presented as percentages and were compared using the chi-squared test. We first determined the incidence per 10,000 of VTE, DVT, and PE found in the IBD cases and controls. To compare the risk of each condition in the cases, relative to controls, Cox proportional hazards model was used to determine crude and adjusted hazard ratios (HRs) [95% confidence intervals (CIs)] after adjusting for potential confounders including presence of events or conditions that provoke VTE and co-morbidities. Next, the incidence rates of VTE were expressed as 1000 person-years, calculated by the number of events divided by the total follow-up (exposed or unexposed) time in each activity state of IBD. Adjusted HRs were calculated with the Cox proportional hazards model with disease activity, hospitalization, and surgery fitted as time-varying covariates, and other covariates as non-time-varying. Covariates included age, sex, recent fracture, pregnancy, and co-morbidities. For evaluating the risks of each activity state of IBD patients, the risk of VTE in controls (non-IBD) was taken as the reference. The HRs comparing flare and non-flare periods of IBD patients with control were first estimated. We subsequently analyzed the effect of disease activity, hospitalization, and surgery in IBD patients on the risk of VTE. We performed a second analysis with data only from IBD patients. In the IBD cohort, adjusted HRs comparing a hospitalized flare, a non-hospitalized flare, a hospitalization without flare with non-flare, and non-hospitalization period were estimated.

In addition, we conducted subgroup analyses to identify interactions between IBD and clinically relevant groups, defined by age (20–59 vs. 60–80 years), sex (male vs. female), disease phenotype (UC vs. CD), diabetes mellitus (no vs. yes), cardiovascular disease (no vs. yes), cerebrovascular disease (no vs. yes), heart failure (no vs. yes), atrial fibrillation (no vs. yes), and chronic kidney disease (no vs. yes). Interactions between subgroups were tested using likelihood ratio tests comparing models with and without multiplicative interaction terms. A *P*-value < 0.05 was considered statistically significant. Statistical analyses were performed using SAS version 9.4 (SAS Institute, Cary, NC).

## Supplementary Information

Supplementary Information.
