# Supplementary material for: Risk of venous thromboembolism in Asian patients with inflammatory bowel disease: a nationwide cohort study
Source: Sci Rep. 2021 Jan 21;11:2025. doi: 10.1038/s41598-021-81657-y (PMC7820465; doi:10.1038/s41598-021-81657-y)
Supplement: Supplementary file 1 — Supplementary Information. [file 41598_2021_81657_MOESM1_ESM.docx]

**Supplementary materialS**

**Title: Risk of venous thromboembolism in Asian patients with inflammatory bowel disease: a nationwide cohort study**

**Short running head:** Venous thromboembolism in IBD

Chan Mi Heo, MD^1^, Tae Jun Kim, MD^1^*, Eun Ran Kim, MD, PhD^1^, Sung Noh Hong, MD, PhD^1^, Dong Kyung Chang, MD, PhD^1^, Mi Yang, MS^2^, Seonwoo Kim, PhD^2^, and Young-Ho Kim, MD, PhD^1^*

**Supplementary Table 1.** Risk of venous thromboembolism in clinically-relevant subgroups

|  | Multivariable-adjusted*  HR (95% CI) | | *P* for interaction |
| --- | --- | --- | --- |
|  | Non-IBD | IBD |  |
| Age |  |  | 0.924 |
| 20-59 (n=27,364) | Reference | 1.97 (1.34-2.89) |  |
| 60-80 (n=5,767) | Reference | 1.99 (1.45-2.74) |  |
| Sex |  |  | 0.011 |
| Male (n=20,118) | Reference | 1.64 (1.14-2.34) |  |
| Female (n=13,013) | Reference | 2.66 (1.89-3.74) |  |
| Disease phenotype |  |  | <0.001 |
| UC (n=23,199) | Reference | 1.74 (1.31-2.32) |  |
| CD (n=9,893) | Reference | 4.43 (2.67-7.34) |  |
| Diabetes mellitus |  |  | 0.84 |
| No (n=29,327) | Reference | 2.11 (1.53-2.89) |  |
| Yes (n=3,804) | Reference | 2.02 (1.37-2.98) |  |
| Cardiovascular disease |  |  | 0.729 |
| No (n=31,430) | Reference | 2.12 (1.59-2.83) |  |
| Yes (n=1,701) | Reference | 1.85 (1.16-2.95) |  |
| Cerebrovascular disease |  |  | 0.817 |
| No (n=32,210) | Reference | 2.00 (1.52-2.62) |  |
| Yes (n=921) | Reference | 2.01 (1.13-3.56) |  |
| Heart failure |  |  | 0.428 |
| No (n=32,672) | Reference | 1.95 (1.50-2.53) |  |
| Yes (n=459) | Reference | 2.56 (1.23-5.34) |  |
| Atrial fibrillation |  |  | 0.454 |
| No (n=32,881) | Reference | 2.06 (1.60-2.66) |  |
| Yes (n=250) | Reference | 1.38 (0.50-3.77) |  |
| Chronic kidney disease |  |  | 0.951 |
| No (n=32,802) | Reference | 1.99 (1.54-2.57) |  |
| Yes (n=329) | Reference | 2.11 (0.82-5.39) |  |

Adjustment for age, sex, provoking factors (recent surgery, fracture, and pregnancy within 90 days of event), and co-morbidities

IBD, inflammatory bowel disease; UC, ulcerative colitis; CD, Crohn’s disease; HR, hazard ratio; CI, confidence interval.

**Supplementary Table 2.** ICD-10 codes for venous thromboembolism

| **Covariates** | **ICD-10 code** |
| --- | --- |
| Deep vein thrombosis | I80, I80.0, I80.1, I80.2, I80.3, I80.8, I80.9, I82 |
| Pulmonary thromboembolism | I26, I26.0, I26.9 |

ICD-10 = International Classification of Diseases, 10th Revision
